# Removal of the Retained Ventricular Catheter Using the Endoscopic Monopolar Instrument

**DOI:** 10.1155/2021/2880979

**Published:** 2021-12-30

**Authors:** Julia Pereira Muniz Pontes, Pedro Henrique Costa Ferreira-Pinto, Elington Lannes Simoes, Thaina Zanon Cruz, Jefferson Trivino Sanchez, Flavio Nigri

**Affiliations:** Department of Surgical Specialties, Neurosurgery Teaching and Assistance Unit, Pedro Ernesto University Hospital, Rio de Janeiro State University, Rio de Janeiro, RJ, Brazil

## Abstract

**Background:**

Ventriculoperitoneal shunt (VPS) remains the main treatment for hydrocephalus. However, VPS revision surgery is very common. Here, we present a case in which the retained ventricular catheter was removed using the endoscopic monopolar instrument.

**Methods:**

We report a case of a 28-year-old female who presented with VPS obstruction. She had two previous shunt revision surgeries due to shunt obstruction. Eleven years after the last one, she presented an abdominal pseudocyst that indicated a total system removal. During VPS revision surgery, a retained ventricular catheter was observed. The endoscopic monopolar instrument was introduced into the retained catheter under direct view. Coagulations in a back-and-forth movement were applied to release inner catheter adhesions. After these steps, the catheter was removed, and a new one was placed through the same route.

**Results:**

The catheter was removed without complications, confirmed by the postoperative cranial computed tomography. The patient remained asymptomatic.

**Conclusion:**

The described technique was effective and avoided ventricular bleeding. Further studies are necessary to validate this method.

## 1. Introduction

Ventriculoperitoneal shunt (VPS) remains the main treatment for hydrocephalus [1, 2]. Approximately 50% of shunts fail, requiring replacement [3]. Removal of the ventricular catheter, when needed, may be risky. Plexus choroid adhesions increase the occurrence of intraventricular hemorrhage [4–7]. Here, we present a case in which the retained ventricular catheter was removed using the endoscopic monopolar instrument. To date, there are no reports indicating the use of this technique according to the medical literature analysis and retrieval system online (MEDLINE) database.

## 2. Case Presentation

**Figure 1:**
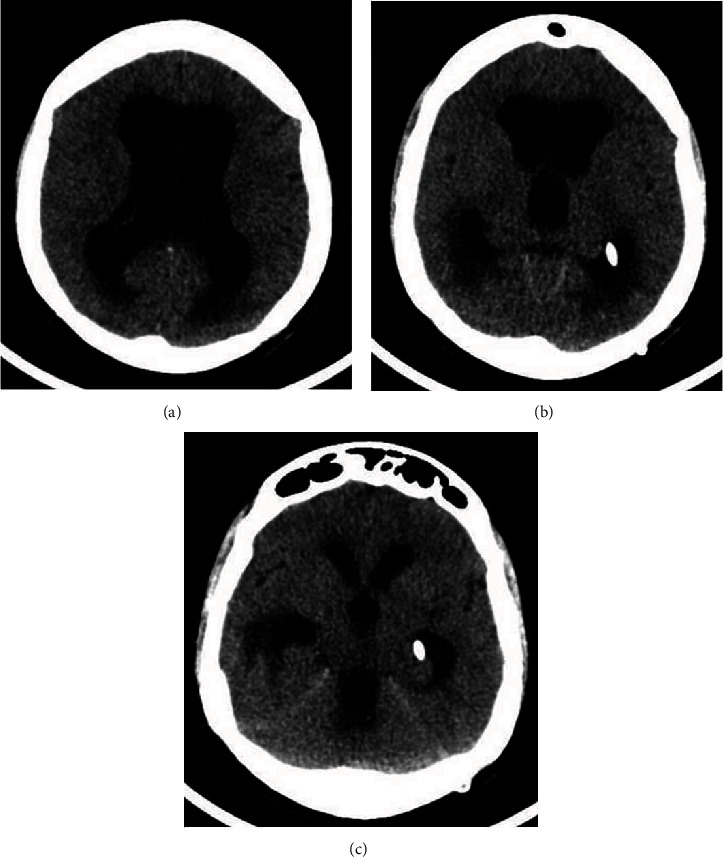
Preoperative cranial CT. (a–c) Axial slices demonstrate ventriculomegaly, enlargement of the temporal horns, and transependymal edema suggesting VPS obstruction. The ventricular catheter is positioned inside the temporal horn.

**Figure 2:**
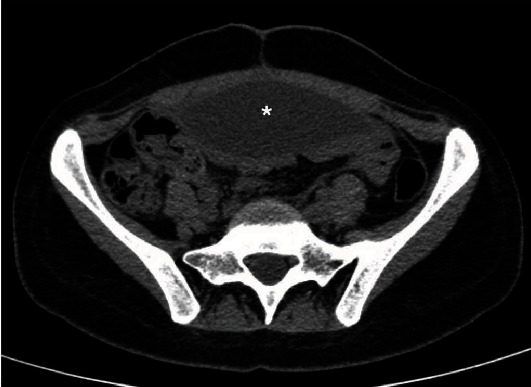
Axial section of the abdominal CT. The asterisk represents the pseudocyst inside the abdominal cavity. This seems to be the cause of the system malfunctioning.

A 28-year-old female patient was referred to our hospital because of headache, vomiting, and abdominal pain. She had her first VPS at the age of 14 due to communicating hydrocephalus secondary to bacterial meningitis. At that time, the ventricular catheter was inserted in the right lateral ventricle through a posterior parietal approach. A shunt revision was performed after two years because of shunt obstruction. The previous system was totally removed, and a new one was placed through a left posterior parietal burr hole. The patient remained asymptomatic for 11 years until the current presentation with symptoms of shunt blockage. The cranial CT scan demonstrated ventriculomegaly, enlargement of the temporal horns, and transependymal edema (Figures 1(a)–1(c)). An abdominal CT scan revealed an oval fluid cystic collection with regular contours. The lesion was compatible with a purely inflammatory abdominal pseudocyst, according to Mallereau et al. classification [8] (Figure 2). The abdominal catheter was exteriorized at the clavicular region and connected to an external ventricular drainage collector. The cerebrospinal fluid (CSF) was normal and sterile. Antibiotic therapy with vancomycin and cefepime for 21 days was administered based on a suspected shunt indolent infection. Three consecutive negative CSF microbiological analyses confirmed that there were no signs of infection. Total removal of the previous shunt was performed because during the procedure of disconnection between the valve reservoir and the ventricular catheter, a low CSF flow was observed due to plexus choroid obstruction. After system removal, a ventriculoatrial shunt (VAS) was performed. A standard left transverse neck incision at the anterior border of the sternomastoid muscle was made, and the linguofacial trunk was dissected out. Then, a subcutaneous tunnel was made from the previous left parietal incision to the site of neck incision using a blunt shunt passer. The distal catheter was passed through the subcutaneous tunnel.

### 2.1. Ventricular Catheter Removal Technique

**Figure 3:**
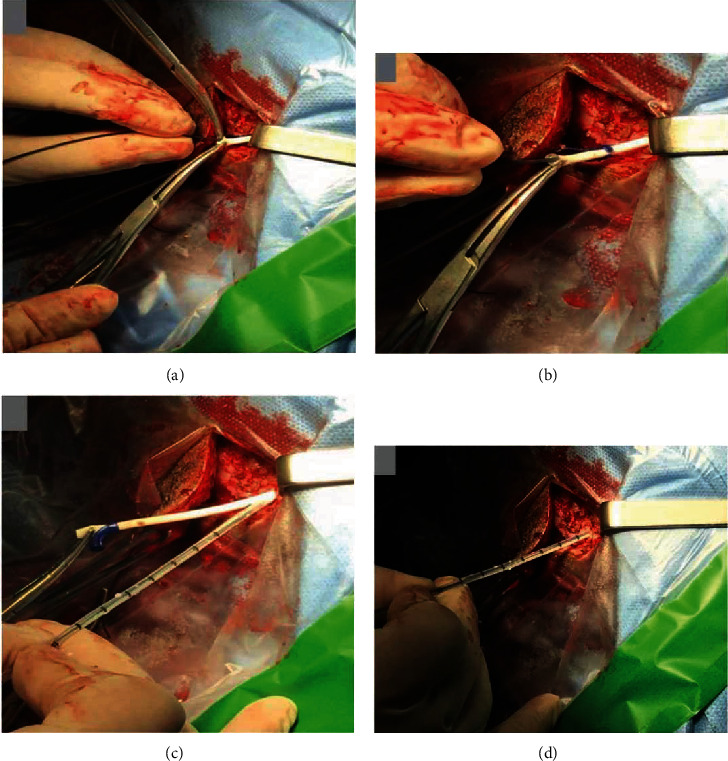
Ventricular catheter removal technique. (a) The monopolar wire being introduced inside the ventricular catheter. Two Kelly forceps were used to fix the catheter. (b) Introduction of the monopolar wire into the existing proximal catheter. (c) Previous catheter removal. The new catheter was positioned very close to the burr hole. (d) The ventricular catheter was inserted using the previous trajectory.

**Figure 4:**
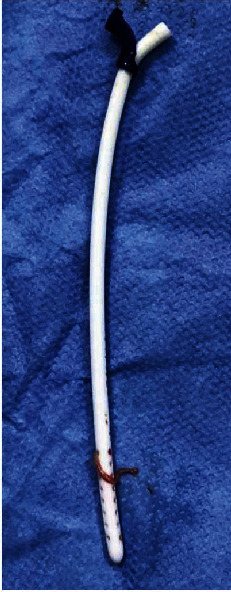
The previous ventricular catheter after removal. This figure demonstrates the choroid plexus adhesions around the original catheter.

**Figure 5:**
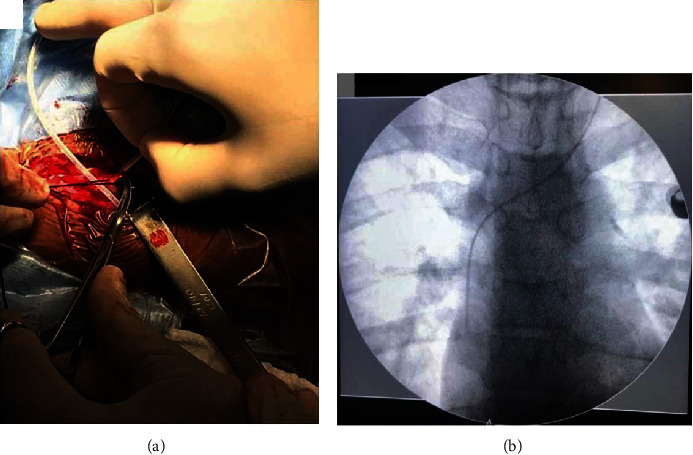
Distal catheter insertion. (a) Insertion of the distal catheter in the right atrium through the left linguofacial trunk. (b) Chest X-ray confirming atrial catheter position.

After cranial exposure, the ventricular catheter was detached from the valve and closed with protected Kelly forceps. It was noted that there was an important resistance to pull out the ventricular catheter. Then, an endoscopic monopolar wire (coagulation electrode unipolar, flexible, diameter 1 mm, length 53 cm, reference number 28160 KA, Karl Storz Gmbh and Co. Kg, Tuttilingen, Germany) was advanced into the ventricular catheter (Figure 3(a)). Great care was taken to prevent CSF leakage. Kelly forceps were used to fix the ventricular catheter in order to avoid pushing it along with the monopolar wire. The monopolar wire was advanced until the end of the ventricular catheter (Figure 3(b)). Then, with the monopolar wire connected to a Valleylab Force FX Electrosurgical Generator (Covidien, Walpole, United States), with power setting at 8 W, a back-and-forth movement was applied to coagulate and liberate inner catheter adhesions. At the same time, a slight pulling force was applied to the ventricular catheter until it was released (Figure 3(c)). Finally, the retained catheter was removed, and a new one was placed through the same route (Figure 3(d)). Adhesions of the choroid plexus passing through catheter holes were identified (Figure 4). The new ventricular catheter was connected to the remaining VAS system (Figure 5(a)) [9]. Then, the appropriate positioning of the atrial catheter was confirmed by fluoroscopy (Figure 5(b)).

### 2.2. Surgery Outcome

**Figure 6:**
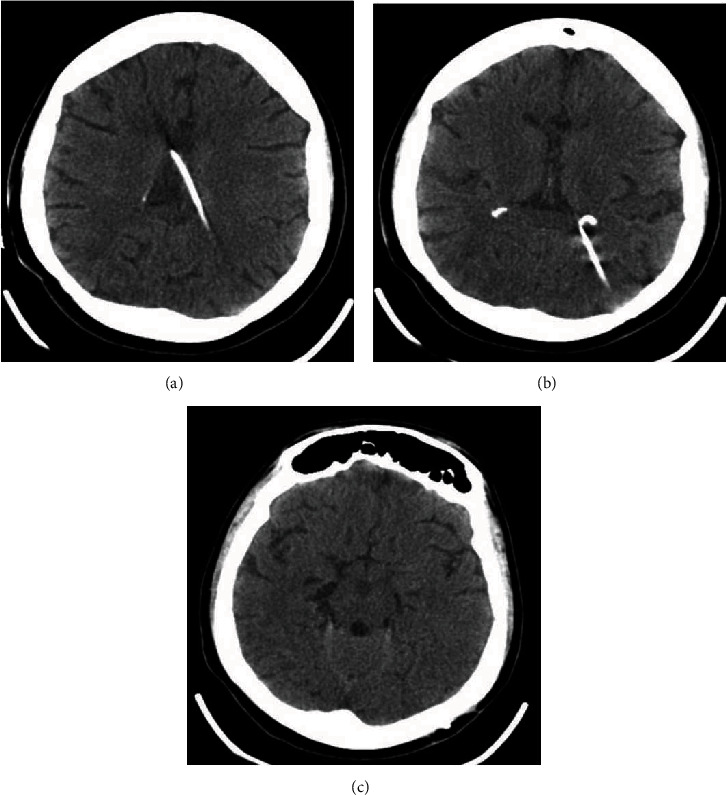
(a–c) Postoperative cranial CT demonstrating the reduction of ventricle sizes. The ventricle catheter is well positioned.

The comparison of the pre- and postoperative cranial CT demonstrated that the new ventricular catheter was functional, well-positioned, and without adjacent bleeding (Figures 6(a)–6(f)). The patient is being followed up for 2 years.

## 3. Discussion

Currently, VPS remains the most performed procedure for hydrocephalus treatment [3, 10]. However, shunt failures and revision surgeries are common [11]. The risk of hydrocephalus and acute intracranial hypertension with fatal outcome will always exist. The reoperations are technically more difficult, and the psychological trauma generated in these patients because of multiple hospitalizations and reoperations cannot be underestimated. The cost of multiple surgeries, replacement of implantable devices, hospitalization, antibiotics, and medications is also considerable.

Pseudocyst formation is a complication of VPS insertion. It is usually caused by shunt blockage, infection, or inflammation [8]. According to Mallereau et al. proposal classification [8], this particular case should be classified as a “purely inflammatory” pseudocyst. In relation to the treatment algorithm proposed by the same group [12], the distal catheter should be repositioned in another abdominal quadrant in cases of sterile inflammatory pseudocysts. However, despite the preliminary negative laboratory and radiological results, we chose to externalize the system and start empirical antibiotic therapy. Only after negative CSF cultures, total removal of the externalized VPS system and a VAS was performed.

In several situations, during shunt revisions, we are obliged to replace the ventricular catheter, mainly because of infection or obstruction. Furthermore, observational studies suggest that partial revision of shunts predisposes to accelerated shunt failure as compared with total revision in cases of an obstructed VPS [10, 13, 14]. The obstruction of the ventricular catheter by ingrowth of the choroid plexus is the most common cause of shunt failure [10]; then, the removal and replacement of the retained ventricular catheter are crucial to obtain an adequate CSF outflow. However, sometimes, it is difficult to remove the ventricular catheter that is attached to the choroid plexus [15] or to other scar tissue. In these cases, when trying to remove the retained ventricular catheter, an intraventricular hemorrhage may occur and can be fatal [1].

To minimize the potential damage from removing the retained catheter, some techniques have been described. Chehrazi and Duncan [16] were one of the first to describe a technique to remove retained ventricular catheter. The retained catheters were pulled through an insulated number 11F or 13F suction tube attached to an electrocoagulation unit used for resection and coagulation of adhesions. Chambi and Hendrick [17] and Whitfield et al. [18] performed a similar method of catheter removal using the catheter stylet. A metal ventricular cannula was inserted into the lumen of the ventricular catheter, and a cutting current from a unipolar diathermy was applied to the distal end. The cutting effect at the interventricular end of the metal cannula effectively lysed the adhesions and allowed safe removal of the ventricular catheter without subsequent bleeding. Percutaneous coagulation of the choroid plexus using the Seldinger technique was utilized by Gnanalingham et al. [19]. Successful removal using an endoscopic view inside [6] or outside the catheter [3, 7] has also been described. Finally, Haldar et al. [5] have successfully included the use of the Valsalva maneuver to release the ventricular catheter.

**Figure 7:**
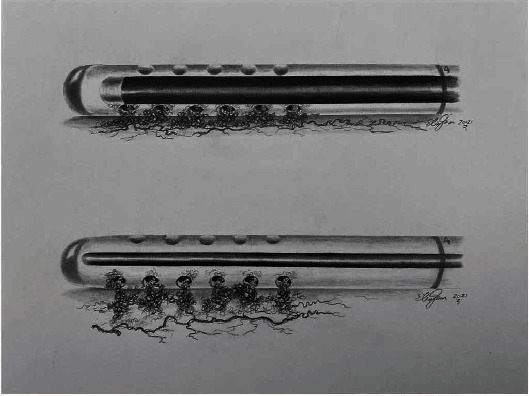
Comparison between endoscopic monopolar wire and stylet techniques. The endoscopic monopolar instrument (top drawing) coagulates the inner content, fibrous tissue and choroid plexus adhesions, releasing the catheter from the adherence, minimizing the risk of any blood vessel rupture during catheter removal. The monopolar wire is larger than the ventricle catheter holes and is semirigid, making it very difficult to go beyond the catheter limits and damage the brain. In the stylet technique, (bottom drawing) the wire, due to its rigidity and size, can pass beyond the catheter limits and damage surrounding brain tissue. Furthermore, the thickness of the stylet does not fill completely the inner space of the catheter, leaving some scar tissue untouched.

The removal of obstructed proximal VPS catheter using the endoscopic monopolar instrument is a therapeutic option when it is difficult to pull out the catheter. The instrument coagulates the inner content, fibrous tissue and choroid plexus adhesions, releasing the catheter from the adherence, minimizing the risk of any blood vessel rupture when the catheter is removed. It is important to mention that the monopolar wire is larger than the ventricle catheter holes and is semirigid, making it very difficult to go beyond the catheter limits and damage the brain. On the other hand, in the stylet technique [17, 18], the wire, due to its rigidity and size, can pass beyond the catheter limits and damage surrounding brain tissue. Furthermore, the thickness of the stylet does not completely fill the inner space of the catheter, leaving some scar tissue untouched (Figure 7). Removal of the adhesions using direct endoscopic view may be a good alternative. However, in some cases, it may be impossible to navigate in small or slit ventricles and also, because of the need of an additional entry point, it may increase morbidity and operative length.

## 4. Conclusion

The presented technique was effective and easy to perform. This procedure can minimize the risk of ventricular bleeding and allows the ventricular catheter to be removed safely. It can be another option in the neurosurgeon armamentarium. Further studies are needed to validate this technique.

## Data Availability

All data generated or analysed during this study are included in this article. Further enquiries can be directed to the corresponding author Julia Pereira Muniz Pontes.

## Abbreviations

et al.: Et alibi
CSF: Cerebrospinal fluid
CT: Computed tomography
EVD: External ventricular drain
ETV: Endoscopic third ventriculostomy
MEDLINE: Medical literature analysis and retrieval system online
mm: Millimeter
VAS: Ventriculoatrial shunt
VPS: Ventriculoperitoneal shunt
W: Watts.
